# Supplementary material for: COVID-19 Severity and Neonatal BCG Vaccination among Young Population in Taiwan
Source: Int J Environ Res Public Health. 2021 Apr 18;18(8):4303. doi: 10.3390/ijerph18084303 (PMC8074147; doi:10.3390/ijerph18084303)
Supplement: Supplementary file 1 [file ijerph-18-04303-s001.zip › ijerph-1160013-supplementary.pdf]

Table S1: Sex distribution in clinical Syndromes associated with COVID-19 stratified by participant's Tokyo-172 BCG vaccinationstatus among age 4-24 years and 25-33 years between January 21 and March 19 2021 in Taiwan.

Female

Birth year	Without neonatal BCG vaccination records						With neonatal BCG vaccination records					
	Asymptomatic	Mild	Moderate	Severe	Critical	Total	Asymptomatic	Mild	Moderate	Severe	Critical	Total
1996-2016	0	2	0	0	0	2	4	57	13	1	1	76
1986–1995	2	38	8	1	0	49	4	34	7	0	0	45
Total	2	40	8	1	0	51	8	91	20	1	1	121

Male

Birth year	Without neonatal BCG vaccination records						With neonatal BCG vaccination records					
	Asymptomatic	Mild	Moderate	Severe	Critical	Total	Asymptomatic	Mild	Moderate	Severe	Critical	Total
1996-2016	0	4	0	0	0	4	11	39	11	1	0	62
1986–1995	5	45	7	0	0	57	2	23	8	0	0	33
Total	5	49	7	0	0	61	13	62	19	1	0	95
